# Sensing developing biofilms: the bitter receptor T2R38 on myeloid cells

**DOI:** 10.1093/femspd/ftw004

**Published:** 2016-01-17

**Authors:** Matthias Martin Gaida, Ulrike Dapunt, Gertrud Maria Hänsch

**Affiliations:** 1 Institute for Pathology, Heidelberg University, Im Neuenheimer Feld 224, 69120 Heidelberg, Germany; 2 Center for Orthopaedics, Trauma Surgery and Spinal Cord Injury, Heidelberg University Hospital Schlierbacher Landstrasse 200a, 69118 Heidelberg, Germany; 3 Institute for Immunology, Heidelberg University, Im Neuenheimer Feld 305, 69120 Heidelberg, Germany

**Keywords:** biofilm, innate immune response, T2R38, bitter receptor, lipid droplets, AHL-12

## Abstract

Quorum-sensing molecules, also known as autoinducer, are essential for bacterial biofilm formation. Our focus is on *N*-(3-oxododecanoyl)-L-homoserine lactone (AHL-12), because it is also known as an ‘interkingdom signalling molecule’, which means that it also interacts with mammalian cells. AHL-12 activates defence-relevant functions of phagocytic cells, including enhancement of phagocytosis, increased expression of adhesion receptors and induction of chemotaxis. This leads to the hypothesis that early recognition of developing biofilms might be the key to a successful host defence against biofilm infection. In that context we studied activation of phagocytic cells by AHL-12, and found that phagocytes are activated via a rather specialized receptor that was not previously described on myeloid cells, the bitter taste receptor T2R38. Taste receptors are commonly associated with cells of the gustatory system. The extragustatory expression, however, suggests an additional role, namely the sensing of the onset of bacterial biofilm infection.

## Abstract (precis)

Myeloid cells express the bitter taste receptor T2R38 als ligand for the quorum-sensing molecule of *Pseudomonas aeruginosa* N-(3-oxododecanoyl)-L-homoserine lactone (AHL-12).

## INTRODUCTION

Phagocytic cells, particularly neutrophils, are extremely well studied as main protagonists of the immediate host response in bacterial infections (reviewed in Witko-Sarsat *et al.*^2000^). They are the first cells to infiltrate an infected site, and numerous bactericidal and cytotoxic mechanisms have been elucidated. The majority of data are derived from studying interactions with free-swimming, planktonic bacteria. However, since the seminal work of Bill Costerton, we know that this is not the only, and may be not even be the preferred lifestyle of bacteria. Rather, bacteria generate biofilms, a major cause of persistent infection especially by opportunists (reviewed in Costerton, Stewart and Greenberg 1999; Arciola, Campoccia and Montanaro 2002). A frequent and reasonable explanation is that living in a biofilm protects bacteria against host defence mechanisms. On the other hand, molecules released from bacterial biofilms, including ‘pathogen-associated molecular patterns’ or ‘danger signals’ attract and activate cells of the innate host defence (reviewed in Jensen *et al.*^2010^; Hänsch 2012). We are especially interested in *N*-(3-oxododecanoyl)-L-homoserine lactone (AHL-12), a quorum-sensing molecule of *Pseudomonas aeruginosa* and of other Gram-negative bacteria (reviewed in Schuster *et al.*^2013^). Aside from mediating interbacterial communication and functioning as an autoinducer, AHL-12 is also known as an ‘interkingdom signalling molecule’, which means that it also interacts with a variety of mammalian cells (Shiner, Rumbaugh and Williams 2005; Cooley, Chhabra and Williams 2008; Holm and Vikström 2014). We and Vikström's group previously described an activation of phagocytic cells by AHL-12, including enhancement of phagocytosis, increased expression of adhesion receptors and induction of chemotaxis. Of note, homoserine lactones with shorter fatty acids or lacking fatty acid did not activate phagocytic cells (Vikstrom, Magnusson and Pivoriunas 2005; Zimmermann *et al.*^2006^; Wagner *et al.*^2007^).

AHL-12 is highly lipophilic, and it has been reported that it partitions into membranes in a receptor-independent manner (Ritchie *et al.*^2007^; Barth *et al.*^2012^). Data analysing transmembrane signals, however, were compatible with a G-protein-linked receptor (Kravchenko *et al.*^2006^; Karlsson *et al.*2012a; Kahle *et al.*^2013^).

Recent data by Tizzano *et al.* (2010) and Lee *et al.* (2012) indicated the bitter receptor T2R38 as a possible receptor for AHL-12 on epithelial cells. T2R38 belongs to a larger family of taste receptors sensing ‘bitter’ which are mainly studied in cells of taste buds (reviewed in Adler *et al.*^2000^; Meyerhof 2005). More recently, we found the expression of T2R38 on peripheral blood leukocytes (Maurer *et al.*^2015^) and in this paper, we examined T2R38 in monocytes and neutrophils in tissue, and monocytic cell lines as well, with special focus on its interaction with AHL-12 and the signalling pathways involved.

## MATERIALS AND METHODS

### Antibodies

The following antibodies to T2R38 were used: bs-86508-A488 (rabbit (rb)IgG, labelled with Alexa Fluor 488, Bioss, Woburn, USA); ab65509 (rb serum); ab 130503 (rb IgG) (both obtained from Abcam, Cambridge, UK); sc-76108 (rb IgG); and sc-34294 (goat IgG) (Santa Cruz, Dallas, USA). Isotype controls were rb IgG labelled with Alexa Fluor (bs-0295P-A488, Bioss, Woburn, USA); rabbit IgG (b176094, Abcam) or goat IgG (sc-3887, Santa Cruz). Rabbit serum was obtained from our animal facility. Secondary antibodies were FITC-labelled anti-rb IgG from goat (111-096-003, Dianova, Hamburg, Germany) or Alexa Flour 488-labelled anti-goat IgG from life technologies (A11055, Darmstadt, Germany). FITC-labelled CD11b and CD66b were from (Beckman Coulter, Germany Krefeld). The antigen peptide, sc-34294P, used to raise the antibody to T2R38 (sc-34294), was purchased from Santa Cruz.

### Patients and biopsies

Tissues from patients suffering from acute infections of the bone and adjacent soft tissue were analysed (n = 8). Standard hematoxylin/eosin (H&E) staining was performed to verify the diagnosis, followed by immunohistochemistry, using anti-human T2R38 (sc-34294, 1:100 and retrieval condition: pH 9.0). As secondary antibody the Dako EnVision kit (Dako) was used, followed by counter stain with hematoxylin. For indirect immunofluorescence analysis, goat anti-human T2R38 (sc-34294, 1:100, retrieval condition: pH 9.0) was used with Alexa488 donkey anti-goat as secondary antibody (Life Technologies, Eugene, Oregon, USA,1:400), as was anti-human CD11b or anti-human CD66b (1:100) and as secondary antibody Cy3-conjugated goat anti-mouse IgG (Jackson Immuno Research, 1:300) were used, followed by nuclear staining with Hoechst dye. Specimens were treated with isotypic IgG (goat and mouse) in comparable protein concentrations, and the respective secondary antibodies in exactly the same dilution were used to set the threshold for the background.

### Neutrophils, monocytes, macrophages and CD34+ stem cells

Blood from healthy volunteers (mainly laboratory personnel and students) was drawn into heparin-coated tubes (Sarstedt, Nümbrecht, Germany) and centrifuged on PolymorphPrep (Axis-Shield, Oslo, Norway), which yields essentially two cell fractions separated according to their size and density. The neutrophil fraction was harvested, washed and resuspended in Hanks balanced salt solution, containing 1% bovine serum albumin (BSA) (Sigma-Aldrich, Steinheim, Germany). This procedure yields more than 90% neutrophils as determined by flow cytometry determining expression of CD66b. The mononuclear cell fraction was also harvested, washed in RPMI (Gibco, Eggenstein, Germany) and placed into plastic dishes to enrich the adherent cells. After 24 hours, the non-adherent cells were removed. Typically, the remaining adherent cells consisted of monocytes (CD14+, 70% and 30% lymphocytes). To generate macrophages, the monocyte-enriched cell fraction was cultivated for 7 days in the presence of interferon gamma (Roche Diagnostics, GmbH, Mannheim, 100 units/ml) and GM-CSF (Promokine Heidelberg, Germany, 50 units/ml). To generate osteoclasts, monocytes were cultivated for 18 days in the presence of M-CSF (R&D Systems, Minneapolis, USA; 25 ng/ml) and RANKL (Preprotech, Hamburg, Germany; 50 ng/ml).

CD34+ stem cells were isolated from bone marrow of patients requiring an autologous bone graft. Bone marrow was harvested from the iliac crest. The aspirate was layered onto a Ficoll gradient, the cell layer was harvested and CD34+ cells were positively selected by antibody-coated magnetic beads (Miltenyi, Bergisch Gladbach, Germany). Informed consent was obtained from the patients and the volunteers, and the study was approved by the local ethics committee.

### Cell lines

U937 and HL-60 were purchased from ATCC (Rockville, MD, USA). The cell lines were propagated in RPMI, containing foetal calf serum (10%), L-glutamine (1%) and penicillin-streptomycin (1%) (all purchased from Gibco). The cells were used in their exponential growth phase.

### AHL-12

AHL-12, biotin-labelled AHL-12 and FITC-labelled AHL-12 were purchased from Cayman Chemical, AnnArbor, MI, USA. As an inactive compound that shares structural features with AHL-12, homoserine lactone (HSL) was purchased from Sigma (96304; *N*-Z-L-Homoserine lactone).

### Cytofluorometry

Cells were subjected to cytofluorometry using FACSCalibur and CellQuest Pro software (Becton Dickinson, Heidelberg, Germany). For intracellular staining, in addition an antibody to T2R38 was used that recognized the c-terminus (ab 130503, abcam, Cambridge, UK). Cells were treated with FACS Permeabilizing Solution 2 (BD Biosciences, New Jersey, USA), preincubated with the Fc-receptor blocking agent and then incubated with the respective anti-T2R38.

### Western blot and pull-down assays

Cells (1 × 10^7^) were lysed with RIPA buffer (Tris-buffered saline containing 1% Nonidet *P*-40, 0.5% sodium deoxycholate, 0.1% sodium dodecyl sulfate, 0.0004% sodium azide, 0.2 M orthovanadate and 0.5 M phenylmethyl-sulfonyl fluoride) and incubated overnight at 4°C with or without AHL-12-FITC (300 μM; Cayman Chemical), then with sepharose beads (Cell Signaling, Danvers, MA, USA). The supernatant was subsequently incubated with an antibody to FITC, coupled to sepharose (2 h at 4°C). Adsorbed fractions were eluted with SDS loading buffer containing β-mercaptoethanol. The samples were applied to a 12% SDS-Gel. Silver staining was performed and western blotting using anti-T2R38 (sc-34294) or an anti-IQGAP (abcam). Secondary antibodies were a POX-conjugated mouse anti-goat IgG (#205-035-108, Jackson Immuno Research, Suffolk, UK, 1:20000). Blots were developed using Amersham Prime Western Blotting Detection Reagent (GE Healthcare, Freiburg, Germany).

### Laser scan microscopy

Cells were placed on cover slips, and fixed with 2% PFA for 15 min. Then anti-T2R38 (6 μg) and the secondary antibody were added (Alexa Fluor 488 donkey anti-goat IgG; life technologies, Carlsbad, USA) to yield green fluorescence or Alexa fluor 633 to yield red fluorescence, and for comparison goat IgG. In parallel, the antibody was added to the cells together with the peptide sc-34294P. To assess AHL-12 binding, either AHL-12-FITC was used or AHL-biotin, the latter detected by streptavidin. The samples were mounted with Moviol (Sigma-Aldrich) and viewed by laser scan microscopy (Nikon) using a 40× objective. For visualization of droplets, Nile red (Sigma-Aldrich) was added (10 μg /ml 20 min, room temperature). Alternatively, an antibody to perilipin (purchased from Biozol, Eiching, Germany).

### Isolation of lipid droplets

Essentially, the method described by Wan *et al.* (2007) was used. In brief, cells were suspended in 3 ml ice cold disruption buffer (25 mM Tris-HCl pH 7.4, 5 mM EDTA, 1 mM EGTA, 0.2 mM PMSF, 50 μg /ml N-α-p-tosyl- L-lysin-chloromethyl-ketone (TLCK), 1 μg /ml leupeptin, 1 μg /ml pepstatin, 1 μg /ml aprotinin) and lysed by nitrogen cavitation (10 min, 800 psi, 4°C). The lysate was mixed with an equal volume of 1.08 M sucrose in disruption buffer without TLCK, centrifuged for 30 min, 1000 g at 4°C to eliminate nuclei and intact cells and then applied to a sucrose density centrifugation. After ultracentrifugation (3.5 h, 34 000 rpm, 4°C), the lipid droplets were in the fraction with 0.54 M sucrose. This fraction was diluted to 0.35 M sucrose and 0.15 M NaCl, overlaid again with 0.27 M, 0.135 M and 0 M sucrose for a second ultracentrifugation step (34 000 rpm, 4°C, 3.5 h). The lipid droplets were now in the fraction with 0.35 M sucrose. Droplets could be recognized by fluorescence microscopy after having incorporated Nile red (see above). For western blotting, the proteins were precipitated with methanol (sample: methanol 1 :4, 5 h,-20°C, followed by centrifugation for 30 min, 4°C, 16 000 g). The pellet was resuspended in a specialized protein sample buffer for SDS-PAGE, so called ‘Laemmli buffer’ (95°C for 10 min; BioRad) and applied to a 12% SDS-Gel. Western blotting was performed as described above.

### Uptake of AHL-12 and inhibition experiments

Cells (1 × 10^6^/ml) in HBSS, containing 1% BSA and 0.1% sodium azide (‘FACS buffer’) were incubated with AHL-12-FITC or AHL-12-Biotin, detected with streptavidin-PE for 30 min at 4°C, and then subjected to cytofluorometry. For inhibition experiments, isolated neutrophils were washed with FACS buffer and incubated with antibodies to T2R38 for 30 min at 4ºC, or with the antigen peptide (2–4 μg), or with HSL. AHL-FITC (100 μM) was added and after 30 min at 4°C, fluorescence associated with the cells was measured.

## RESULTS

### Expression of T2R38 on leukocytes

**Figure 1.**
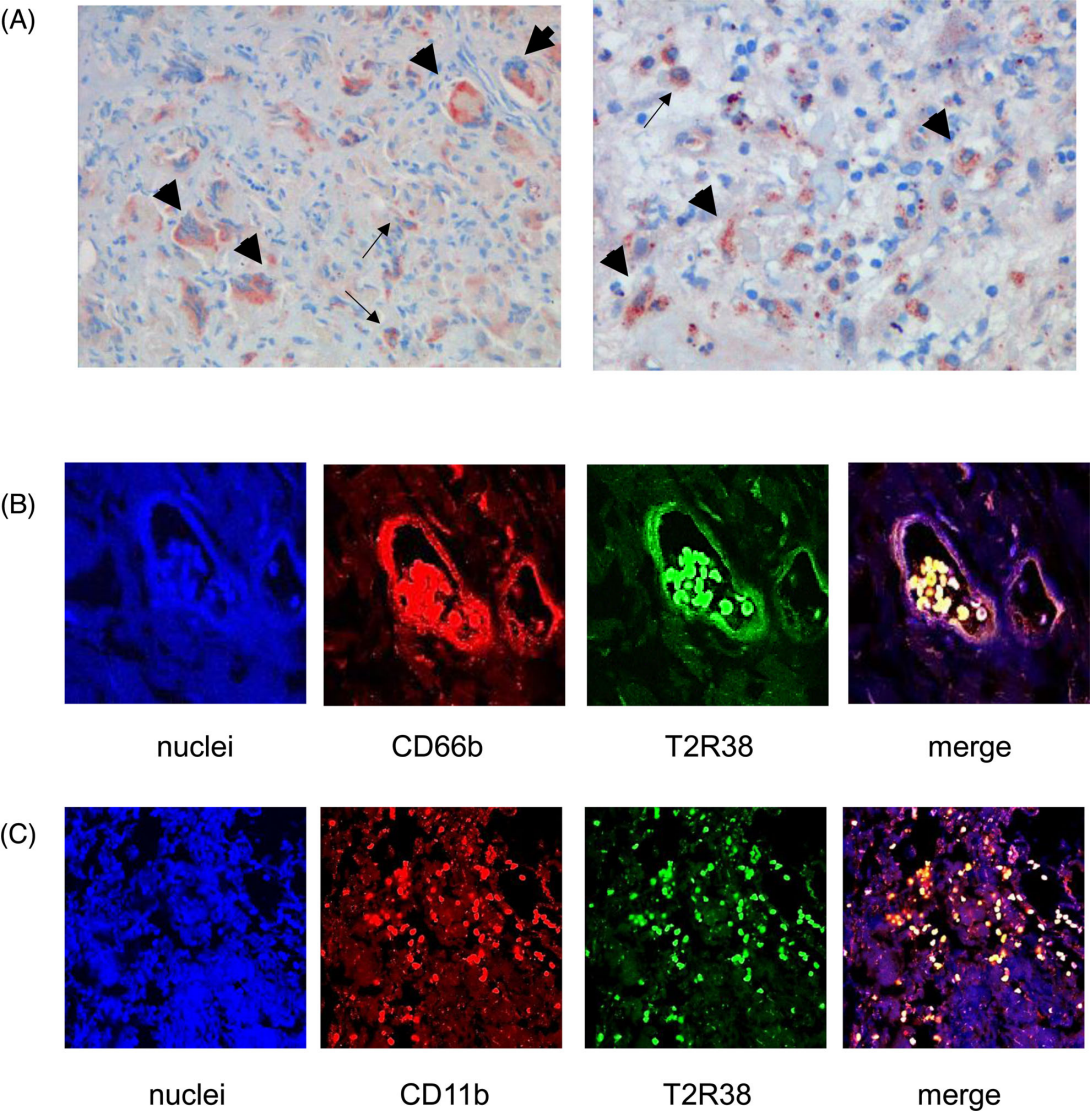
*Expression of T2R38 in tissue*: (**A**) T2R38 expression (brown colour) was seen in biopsies of patients with bacterial infection in association with infiltrated cells (arrows) and bone-resorbing osteoclasts (arrowheads). Unstained cells appear blue. (**B**) By indirect immunofluorescence, T2R38 expression on neutrophils attached to a blood vessel was seen, and in (**C**) on infiltrated CD11b positive cells (neutrophils, macrophages).

In biopsies from patients with bacteria-induced osteomyelitis, prominent expression of T2R38 was seen on the infiltrated phagocytic cells, for example in neutrophils being attached to a blood vessel, in the infiltrated neutrophils and macrophages throughout the tissue, and in multinucleated osteoclastic giant cells as well (data of two patients are shown in Fig. 1).

By cytofluorometry and laser scan microscopy, T2R38 was also detected in peripheral blood cells, including neutrophils, monocytes and lymphocytes, and the myeloid cell lines HL-60 and U937. All cells expressed T2R38 on the membrane and intracellularly.

**Figure 2.**
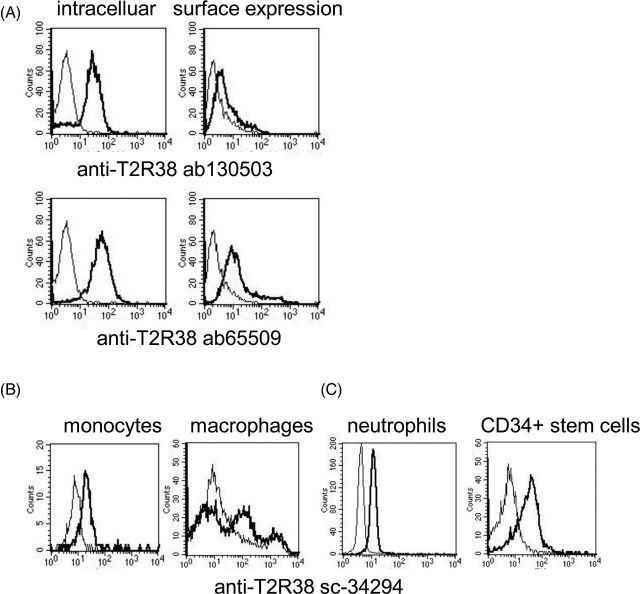
*Expression of T2R38 on myeloid cells*: (**A**) By cytofluorometry, T2R38 was determined on HL-60 cells. By use of an antibody specific for the C-terminus of the receptor (ab130503), T2R38 was detected intracellularly; by an antibody detecting an epitope near the N-terminus (ab65509), T2R28 was seen inside and on the cell membrane (thick line: antibody; thin line rabbit serum as isotype control). (**B)** Monocytes express T2R38 on the surface; upon differentiation to macrophages surface expression is prominently increased. (**C)** T2R38 is also found on isolated neutrophils and on isolated CD34+ stem cells (in B and C anti-T2R38, sc-34295 was used; thick line; the thin line shows goat IgG as isotype control).

Of note, surface expression of T2R38 on monocytes increased prominently when monocytes were differentiated to macrophages (Fig. 2).

Because these data implied that expression of T2R38 is an early trait of myeloid cells, we assessed CD34+ stem cells, and we also detected T2R38 intracellular, and on the membrane (Fig. 2).

### Intracellular storage of T2R38

**Figure 3.**
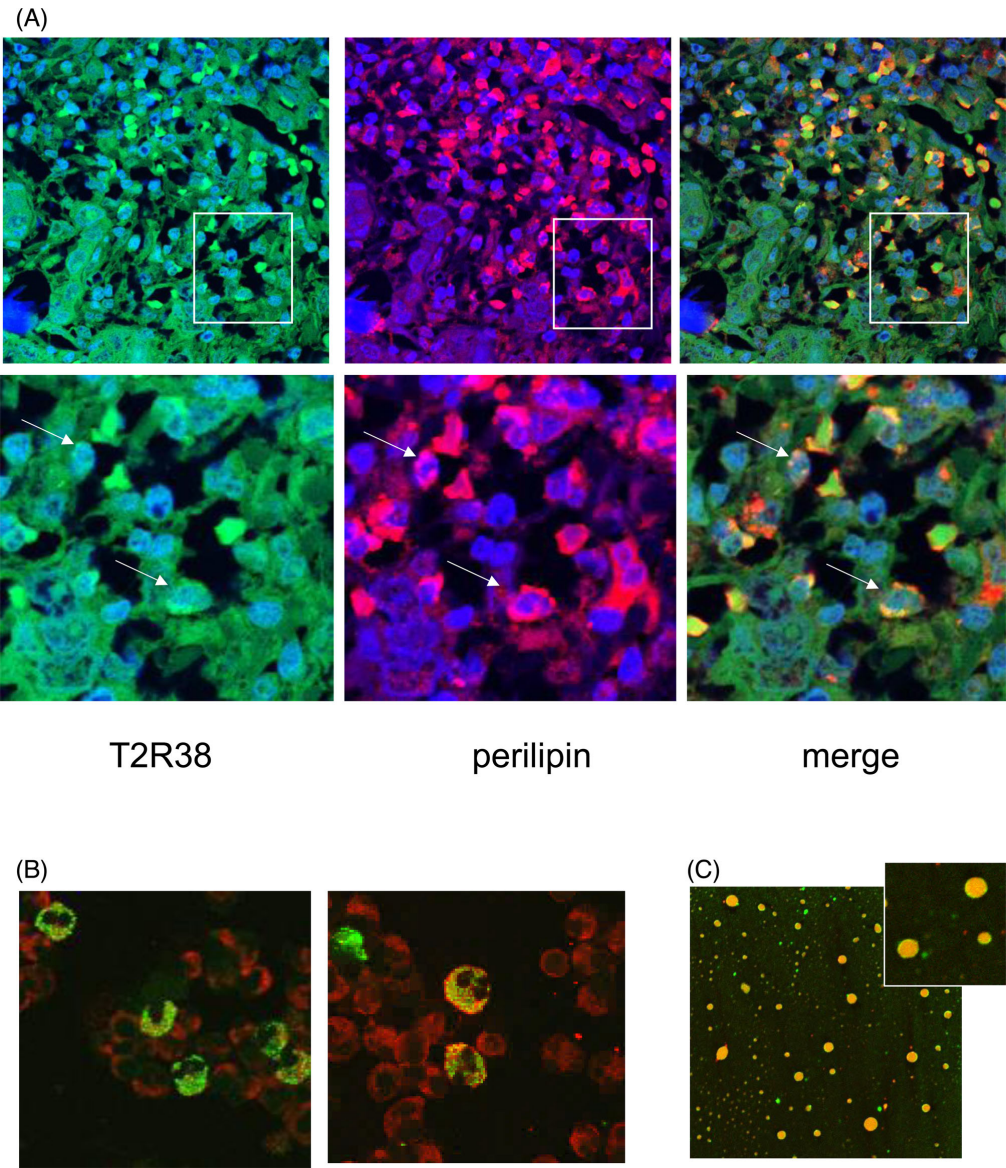
*Association of T2R38 with lipid droplets*: (**A**) Cells in biopsies were stained with anti-perilipin to detect lipid droplets (red), and with anti-T2R38 (green). The merged images show an overlay of the two colours (yellow), indicative a close vicinity of T2R38 and lipid droplets. The upper panel shows the magnification (40×); the lower panel a digital zoom of a selected area. (**B**) Shown are isolated neutrophils, T2R38 shows a speckled pattern (left image, green); on the right, lipid droplets were stained with Nile red, T2R38 in green and an overlap of the two colours is seen (yellow dots). (**C**) Droplets were isolated from HL-60 cells; they were heterogeneous in size, stained with Nile red and T2R38 (green) was found on the droplet membrane.

Laser scan microscopy revealed a T2R38 expression in a speckled pattern, indicative of storage in vesicles. These were identified as lipid droplets, due to their content on perilipin that could be detected with an antibody, and by staining with Nile red. T2R38 co-localized with lipid droplets, as seen by examining tissue as well as isolated cells (Fig. 3). Moreover, isolation of lipid droplets from cells (HL-60 and U937) confirmed the presence of T2R38 and an association with the lipid droplet membranes (Fig. 3).

### Association of AHL-12 with T2R38 and IQGAP

AHL-12 binds to cells, as shown by cytofluorometry using either FITC-labelled AHL-12 or biotin-labelled AHL-12. Binding could be inhibited by HSL, which shares structural features with AHL-12, but is not biologically active (Fig. 4). Binding of AHL-12, however, could not be inhibited by any of the antibodies to T2R38. These experiments do not rule out T2R38 as a receptor for AHL-12, because previous data by others using different experimental approaches had reported that the commercially available antibodies did not block epitopes of the receptor relevant for ligand binding (Behrens *et al.*^2012^).

**Figure 4.**
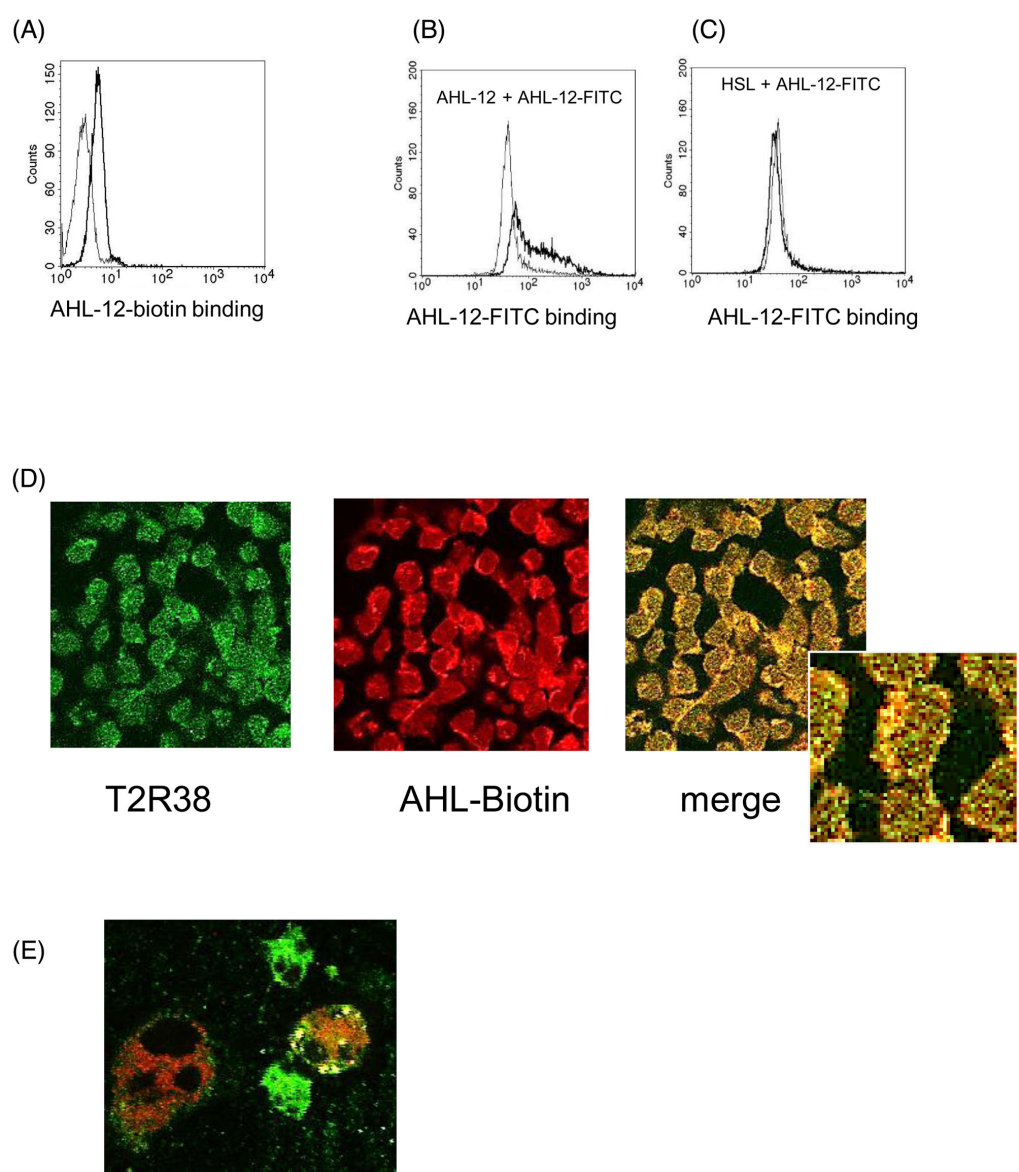
*Binding and association with T2R38, AHL-12 and IQGAP*. (**A**) Biotinylated AHL-12 or (**B** and **C**) FITC-labelled AHL-12 bind to cells (in this example to U937), and binding of AHL-12-FITC could be inhibited by AHL-12, but not by HSL (AHL-12-FITC thick line; following preincubation with wither AHL-12 (in B) or HSL (in C) thin line. (**D**) By laser scan microscopy, T2R38 (green) was seen within the cell (U937) in a speckled pattern; AHL-12-biotin (red) staining was more diffuse, and the merged image shows co-localization (yellow dots), particularly well visible on the digital zoom. Panel (**E**) shows the merged image of a similar experimental approach as in (D), but with neutrophils. (**F**) The pull-down assay with AHL-12-FITC as bait yielded numerous bands (seen by SDS-PAGE and silver staining); those differentially expressed in AHL-12-FITC-antibody versus random-antibody-coated beads are marked by arrows. Western blot carried out in parallel, identified one of the bands as IQGAP, the other as T2R38. (**G)** Expression of IQGAP (red) was associated with that of T2R38 (green), yielding yellow staining, particularly obvious on polarized cells (images of various cells are shown; from left to right neutrophil, migrating neutrophil, monocyte, osteoclast).

**Figure 4.**
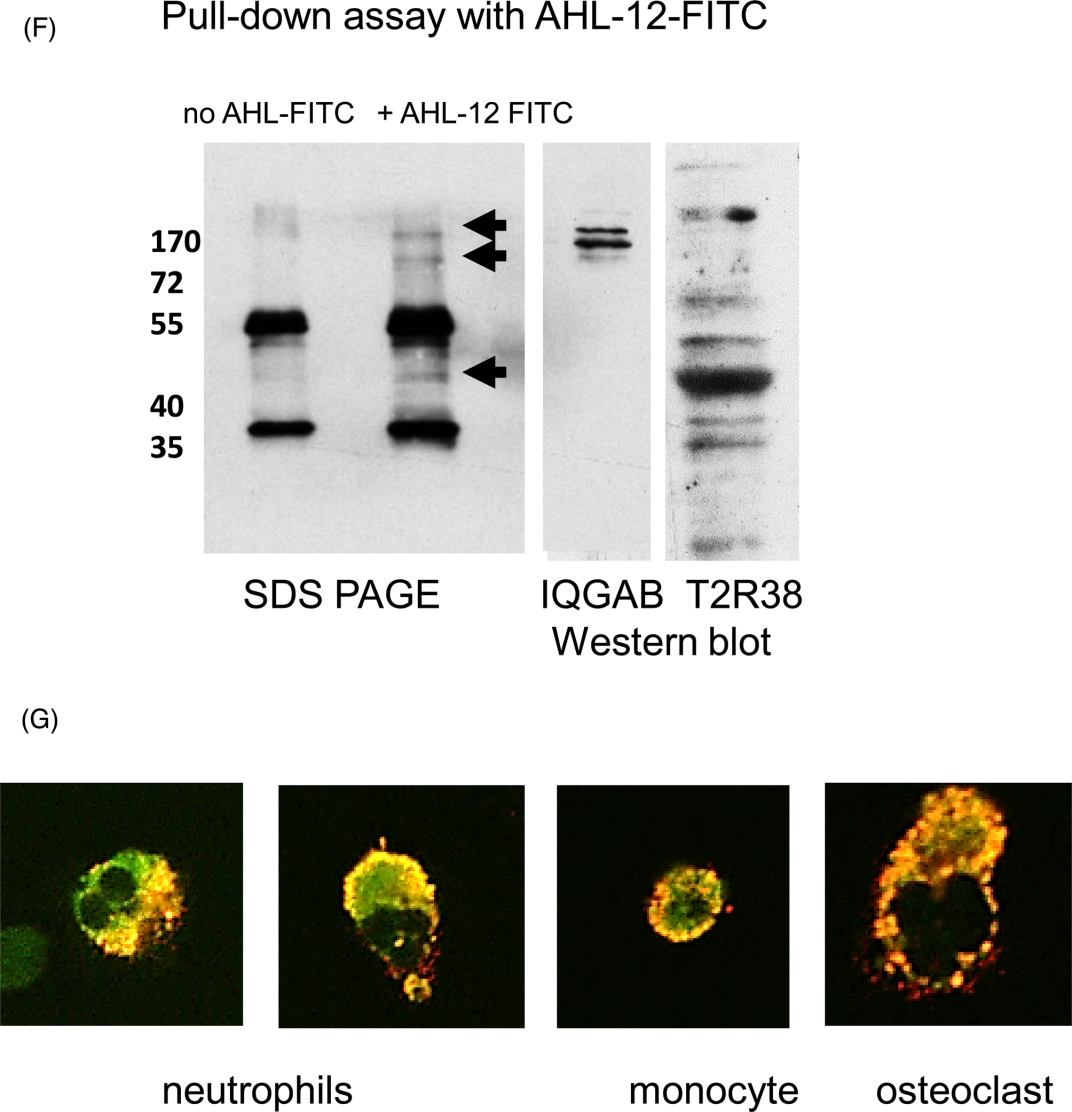
(*Continued*).

Co-localization of AHL-12 with T2R38 was assessed by laser scan microscopy. U937 or neutrophils were incubated with AHL-12-FITC or AHL-12 biotin, fixed and T2R38 was visualized using an antibody. As seen in the example in Fig. 4, there was an overlap between AHL-12 and the receptor, indicative of a close spatial relationship. To confirm this relationship, an antibody to FITC was immobilized to sepharose beads, and incubated with lysates of U937 that had to be incubated with AHL-12-FITC. The adsorbed material was eluted and examined by SDS-PAGE. Multiple proteins appeared, and compared to lysates that had been adsorbed to sepharose beads carrying a random IgG, a few were differentially expressed (marked on the example in Fig. 4F). By western blotting, one of those bands was identified as T2R38, and another as IQGAP, the latter consistent with a previous report by Vikströms group (Karlsson *et al.*2012b). By laser scan microscopy, we could also show a co-localization of T2R38 with IQGAB, particularly in activated, polarized neutrophils showing a polarization, indicative of activation (Fig. 4G).

## DISCUSSION

In this study, we identified the bitter receptor T2R38 as a novel receptor for the quorum-sensing molecule N-(3-oxododecanoyl)-L-homoserine lactone (AHL-12) on myeloid cells. These findings answer one of the most pertinent questions, since AHL-12 was recognized as an ‘interkingdom signalling’ molecule (reviewed in Cooley, Chhabra and Williams 2008; Hansch 2012). Although numerous data pointed to a receptor-mediated activation of mammalian cells, there was the observation that AHL-12, due to its lipophilic nature, can partition into cells independent of a receptor (Barth *et al.*^2012^). More recent studies, however, identified the bitter receptor T2R38 on epithelial cells as receptor for AHL-12 (Tizzano *et al.*^2010^; Lee *et al.*^2012^).

T2R38 belongs to a large family of chemosensory receptors, which were initially detected in cells of taste buds. Numerous ligands, particularly bitter tasting food constituents, have been identified as ligands. In recent years, a broader extragustatory distribution of some of the bitter receptors was reported, for example, in breast cancer cell lines, enteroendocrine cells of the colon, neutrophils or airway epithelial cells (Wu *et al.*^2002^; Rozengurt 2006; Jeon, Seo and Osborne 2011; Singh *et al.*^2011^, ^2014^; Lee *et al.*^2012^; Maurer *et al.*^2015^).

We now detected T2R38 in biopsies of patients with bacteria-induced osteomyelitis, particularly associated with infiltrating leukocytes and osteoclasts. The histologic images suggested that T2R38 was mainly located in the cytoplasma in close association with lipid droplets. These data could be confirmed with leukocytes isolated from the peripheral blood of healthy donors, and myeloid cell lines. Flow cytofluorometry showed intracellular expression of T2R38, as well as an association with the plasma membrane. Laser scan microscopy confirmed the association of T2R38 with droplets, more specifically with the droplet membrane.

Lipid droplets were initially described as mere storage compartment for excessive fat. More recent studies, however, showed that they are functional organelles. By proteomic studies, a multitude of proteins has been found in lipid droplets, particularly those associated with lipid turnover, intracellular trafficking and protein synthesis (Wan *et al.*^2007^). It has been suggested that lipid droplets are an interim storage compartment for proteins awaiting posttranslational modification, particularly for those predestined for binding in a lipophilic environment, as it is the case for T2R38 (reviewed in Welte 2007; Farese and Walther 2009; Beller *et al.*^2010^).

T2R38 is a G-protein coupled receptor, and the signalling pathway described for AHL-12—though in ignorance of a receptor—concurs with G-protein-dependent signalling, (Kravchenko *et al.*^2006^; Karlsson *et al.*2012a; Kahle *et al.*^2013^). Signalling via a G-protein linked receptor is also in line with our finding of an association of T2R38 with IQGAP1. IQGAP1 acts as a scaffolding protein, which by integrating signalling molecule cascades, participates critically in the rearrangement of the cytoskeleton and in regulating cell morphogenesis. Among others, it is a key regulator of chemotaxis, which agrees with the observation that AHL-12 induces chemotaxis. As far as it is known, all chemokines induce chemotaxis via G-protein-linked ‘seven transmembrane receptors’; therefore, it is reasonable to assume that also AHL-12 uses this pathway.

Of note, although the signalling data and the functional studies concur with T2R38 as a membrane-bound surface receptor, intracellular receptor binding cannot be excluded. AHL-12 is highly lipophilic, and partitions into cell membranes in a receptor independent manner, as shown with artificial membranes (Jakubczyk *et al.*^2012^). Moreover, free diffusion of AHL-12 into cells has been described for lymphocytes (Ritchie *et al.*^2007^), and peroxisome proliferator-activated receptors have been proposed as an intracellular receptor (Jahoor *et al.*^2008^). That a ligand uses two independent signalling pathways has been the case for other mediators, for example for leukotriene B (Narala *et al.*^2010^).

The biological role of T2R38 beyond tasting ‘bitter’ is still under investigation. There is, however, strong evidence that via binding AHL-12 T2R38 participates in the local host defence. Engaging T2R38 on airway epithelial cells induces bactericidal mechanisms; moreover, receptor allotypes with low AHL-12 binding capacity predispose to infection (Lee *et al.*^2012^; comments in Viswanathan 2013). Furthermore, the expression of T2R38 on phagocytic cell and the fact that AHL-12 induces or upregulates defence-associated functions could be crucial for the local defence against developing biofilms. Because AHL-12 as quorum-sensing molecule is produced and released by the bacteria as early signal for biofilm formation, it will simultaneously attract phagocytic cells to the site of the developing film; because planktonic bacteria are more susceptible to phagocytosis and bactericidal entities, bacteria might be cleared before manifestation of the biofilm. Thus, by ‘sensing’ bacteria in critical concentration, T2R38 can be viewed as a novel though exceptional ‘pathogen-associated molecular pattern’ receptor.
